# Caregivers’ Beliefs and Stress Among Rural and Urban African American Chronically Ill Caregivers

**DOI:** 10.1093/geroni/igaa057.1092

**Published:** 2020-12-16

**Authors:** Idethia Shevon Harvey

**Affiliations:** Texas A & M University, Bryan, Texas, United States

## Abstract

Introduction: African American women bear a disproportionate burden of chronic disease and disability compared to non-Hispanic whites, particularly rural African American women. The number of African American women providing informal care is increasing, and constant stress among caregivers produces long-term effects among middle-aged and older individuals. The objective of this study was to examine the relationship between stressors and attitudes of caregiving among chronically-ill African American rural and urban women. Methods: The sample included 519 rural and urban African American female caregivers (M = 53.8 years; SD = 15.05), with at least one chronic condition, participated in the Black Rural and Urban Caregivers Mental Health and Functioning Study. Descriptive statistics, Pearson correlation, and hierarchical linear regression analyses were performed among rural women with chronic conditions (N=265) and urban women with chronic conditions (N=254). Results: The number of chronic diseases was the most significant predictors, explaining 16% of stress, followed by caregiver beliefs, socio-demographic factors, and caregiving related factors (F = 21.50, p < 0.01). Discussion: The findings from this study can provide rural health care providers and rural health educators with a basis to assess potential support and disease management programs among chronically-ill caregivers.

